# Cases of Small-Pox after Vaccination; with Remarks

**Published:** 1824-09

**Authors:** James Hardy

**Affiliations:** Member of the Royal College of Surgeons, London; and Member of the Sheffield Medical and Surgical Society.


ART. IV.—*Cases of Small-Pox after Vaccination; with Remarks.* By JAMES HARDY, Esq. Member of the Royal College of Surgeons, London; and Member of the Sheffield Medical and Surgical Society.

THE occurrence of small-pox after vaccination is a subject which, from its importance, has attracted the attention, not only of the medical profession, but of the world in general; and, although the subject has been treated in a very copious and scientific manner, it is far from being exhausted.

There exists in the public mind a very great difference of opinion respecting the propriety or efficacy of vaccination: indeed, the opinions of members of the medical profession are far from being unanimous on the subject; and, whilst that is the case, there is little prospect of the public mind becoming so. Under these circumstances, I think it the duty of every one to communicate such facts as may have fallen under his observation, which may have a tendency to elucidate the subject.

Having lately had the fortune to meet with several cases of this disease, it has induced me to choose this as the subject of my paper. Indiscriminate or extravagant praise, although it may temporarily succeed in establishing a remedy, will, unless it be founded in truth, be sure to be detected in the end, and injure the cause it was intended to uphold. Although, perhaps, a great majority of the profession (and amongst which I must be allowed to rank myself,) advocates the cause of vaccination, yet I do not conceive the subject established on such an immutable basis as to preclude any change of opinion. It is justly remarked by Dr. Gregory, in a valuable paper which appears in the last volume of the Medico-Chirurgical Transactions, that these unpleasant occurrences are on the increase, and no certainty exists that they have yet reached their maximum. I have for a long time wished to satisfy myself, whether vaccination is capable of affording different degrees of protection in the same subject. Dr. Gregory, if I understand him correctly, unhesitatingly decided in the affirmative. Undoubtedly, we witness occasionally after vaccination every degree which small-pox is capable of exhibiting; yet how are we to decide whether this be owing to the degrees of protection afforded, or to peculiarity of constitution in the patient? Vaccination may have gone through its course in perfection in both cases, not only in appearance, but in reality; it may have produced the greatest effect which it is capable of producing, and yet, in some constitutions, not be a preventive of small-pox.

Most writers, who have treated the subject, lay great stress upon the appearance of the cicatrix: as far as my experience enables me to judge, much reliance is not to be placed upon this circumstance. I have witnessed the disease in nearly every degree, under almost every appearance of the cicatrix, and I am inclined to think that, in general, when vaccination produces a pustule containing virus capable of reproducing the same disease, it has effected all which it is capable of effecting.

Case I. March 3, 1824.—B. M. aged thirteen years. She has been vaccinated; the cicatrix was distinct, about middle size, and slightly cellular. She had very high fever, with delirium; the eruption soon became vesicular, and became pustular about the seventh or eighth day of the disease: it was very numerous on the arms, but moderate on the face and body. On the ninth day, they began to decline very rapidly, and the fever had quite subsided. On the sixth, seventh, and eighth days, there was considerable swelling. On the thirteenth day, I found her playing in the street. The pocks had almost all disappeared, and there was no secondary fever.

Case II. March 7, 1824.—M. aged one year, was vaccinated at the Infirmary, Sheffield, about four months ago: the pustules (two) were very much inflamed, and sore for a long time; in fact, they are only just got well: they suppurated several times; they are now of a moderate, not large size,—not cellular. The disease was very mild in this case; the eruption was small, and not numerous, except about the knees and one or two other places: they all died away about the third or fourth day. It was attended by very little fever.

Case III. March 14, 1824.—G., about three years of age, was vaccinated at the Infirmary about a year ago: the cicatrix is of the common size, rather faint; and his parents state that he had a very fine pock, which went through the regular course. He is now under the influence of small-pox (the ninth day): the pustules are very numerous, though generally distinct, and they do not appear to be at all modified. The fever is, perhaps, rather less than might be expected.

April 2.—The pox continued quite as long as in the natural disease.

Case IV. March 13, 1824.—M., fifteen years of age, sister to the subject of Case II., has been vaccinated: cicatrix middle size, faint.

21st.—She has had the disease very slightly: the pustules exceedingly small, not numerous, and disappeared in four or five days. The general indisposition was but slight.

Case V. March 20, 1824.—B., nine years of age, sister to the subject of Case I.: cicatrix similar to Case IV.; considerable fever; the pustules very few and small.

26th.—She was well in a few days.

Case VI. April 2, 1824.—D. H., betwixt two and three years of age, has been vaccinated, and there are two distinct moderate-sized cicatrices. An eruption of small-pox came out on the 27th, 28th, and 29th of March, numerous, but distinct: they are rather smaller than in the natural disease, but appear to be running through the regular stages. The fever was very high, and the patient died on the 10th of April.

There have been many cases of small-pox in unprotected children, in the immediate neighbourhood of this case and of case 3d; and yet it is a singular fact, that these two cases were more severe than any of the others; a circumstance which, it is natural to suppose, would have a tendency to prejudice the minds of those acquainted with it against vaccination.

Case VII. April, 1824.—L. W., nine years of age, was vaccinated at the Infirmary, when an infant: there were two distinct cicatrices on the arm. The eruption was moderate, and began to decline in about eight days.—A sister of the above, three years old, and who was unprotected, received the small-pox infection from her brother, and had the disease rather mildly.

Case VIII. May, 1824.—L. I., a brother to the subject of Case VII., was vaccinated by a woman in the neighbourhood: has a distinct and cellular cicatrix. He had considerable fever, and a slight eruption, for a day or two.

Case IX. March 17, 1824.—Mrs. F., twenty-six years of age, had the small-pox naturally when a child, and some of the cicatrices are very distinct. Fever commenced on the 14th, is very violent, and she has had a convulsive fit to-day, and is sometimes delirious. An eruption, similar to small-pox, is making its appearance.

19th.—The eruption has made the usual progress, is not very numerous, and the fever is considerably abated. The skin surrounding the pocks is, perhaps, more inflamed than usual.

25th.—The disease appears to be scarcely, if at all, modified.

Case X. May 17, 1824.—Samuel F., three years old, son to Mrs. F., was vaccinated at the Infirmary this day week; has had a fit to-day, with symptoms similar to his mother's, with an eruption. The vaccine vesicles are small, and appear to be not at maturity.

19th.—He is better: the eruption is more numerous than that of his mother.

25th.—There appears to be no difference between this disease and natural small-pox.

CASE XI. May 17, 1824.—F., another child of Mrs. F., four months old, was vaccinated at the Infirmary this day week: has slight fever, and a trifling eruption. The vaccine vesicles are arrested in their progress; they appear as they usually do on the fourth or fifth day.

25th.—The eruption never came to maturity, but remained small and shrivelled, with an increase of fever. The child died this morning.

CASE XII. May 26, 1824.—L. E., seven years of age, sister to the subject of Case VII. &c. She was vaccinated at the Infirmary when one year old: there is a fair and distinct cicatrix on the arm. She is now very full of small-pox: they came out on the 19th, and appear to be unmodified; they are of the distinct kind, but very numerous.

CASE XIII. June 5, 1824.—S., aged three years, has been vaccinated at the Infirmary; has a brother and a neighbour labouring under small-pox. An eruption came out this morning.

12th.—Disappeared in a few days.

I saw another case at the same time, closely resembling the above; and another since.

Dr. Gregory says, that "small-pox after vaccination unquestionably prevails in particular families, showing that in them there exists some peculiar susceptibility of the variolous poison. Various instances of the kind have fallen under my own immediate observation."

It will be perceived, from the cases which I have related, that the same circumstances occurred to me; but, in my opinion, the circumstance of exposure is a more probable explanation of the fact than the one given by Dr. Gregory; particularly as the cases given by him in illustration, as well as those related by me, occurred nearly at the same time, and in subjects of very different ages.

*Sheffield; June 16th,* 1824.

